# Comparative analysis of color stability in preheated vs. non-preheated injectable composites under different curing protocols: an in-vitro study

**DOI:** 10.1186/s13104-026-07646-4

**Published:** 2026-02-09

**Authors:** Karthik Shetty, Ashwini Anand Kamat, Roma Mascarenhas, Heeresh Shetty, Laxmish Mallya, Annapoorna Shenoy

**Affiliations:** 1 Department of Conservative Dentistry and Endodontics, Manipal College of Dental Sciences Mangalore, Manipal Academy of Higher Education, Manipal, 576104 Karnataka India; 2 Department of Conservative Dentistry and Endodontics, Nair Hospital Dental College, Mumbai, Maharashtra India

**Keywords:** Resin-based composites, Colour stability, Preheating, Light-curing units, Coffee staining

## Abstract

**Objective:**

This in vitro study aimed to evaluate the effect of composite preheating and light-curing mode on the colour stability of a nanohybrid and a nanofilled resin-based composite after exposure to a controlled coffee-staining protocol. The data presented represents a single observation and is not a part of another research project.

**Results:**

Eighty-disc shaped specimens (10 mm × 2 mm) fabricated from G-aenial Universal Injectable and Filtek Supreme Flowable were assessed under different preheating and curing protocols, subdivided into 8 subgroups. All specimens exhibited colour changes exceeding the clinically perceptible threshold (ΔE > 2.7) after staining. Mean colour change values ranged from 12.0 to 26.5. G-aenial Universal Injectable showed significantly greater discolouration than Filtek Supreme Flowable (*p* < 0.001). Polywave curing resulted in higher mean ΔE values compared with monowave curing (*p* < 0.001). Preheated specimens demonstrated greater colour change than non-preheated specimens for both materials. The highest discolouration occurred in the G-aenial preheated polywave subgroup (ΔE = 26.5), while the lowest occurred in the Filtek preheated monowave subgroup (ΔE = 12.0). Composite formulation, preheating, and curing mode were found to significantly influence colour stability following simulated coffee exposure.

## Introduction

Resin-based composites are widely used in restorative dentistry for their aesthetics, handling, and mechanical performance across diverse indications [1]. Their long-term success depends not only on physical endurance but also on optical stability, since colour change undermines restoration appearance, reduces patient satisfaction, and can necessitate premature replacement of otherwise functional restorations [2, 3].

Discolouration of composite resins arises from intrinsic factors, such as matrix degradation and photoinitiator instability, as well as extrinsic influences, including dietary chromogens and oral environmental exposure, which together accelerate noticeable colour changes [4].

Modern composites such as nanohybrid and nanofilled composites with improved resin matrices and advanced filler architectures improve long-term optical stability. While nanofilled composites only use nanoscale particles and nanoclusters, nanohybrid composites blend micro- and nano-sized fillers [5, 6]. These differences in filler chemistry and distribution are reported to enhance their mechanical performance, optical qualities, and polish retention. However, as composite resins are susceptible to discolouration due to a multitude of factors such as the resin chemistry, filler dispersion, degree of conversion, water sorption, etc. different composites exhibit unique and material-dependent discolouration patterns under different staining conditions [7–10].

Coffee is a potent extrinsic chromogen that penetrates polymer networks and alters colour, even on polished composite surfaces [6, 11, 12]. Immersion studies show that coffee exposure can exceed the clinical acceptability threshold [13, 14], making it a reproducible model for simulating long-term staining in-vitro [15].

Preheating composites before placement has been promoted to improve adaptation, handling, and degree of conversion, ensuring long-term durability [16, 17]. However, its influence on colour stability remains inconclusive, and the available literature on this aspect is limited [18, 19].

Preheating resin composites alters polymerisation dynamics by reducing viscosity and enhancing monomer mobility, resulting in faster polymerisation rates and higher degrees of conversion compared with room-temperature materials. However, the accelerated reaction may advance the gel point and influence shrinkage stress development [19]. Evidence suggests that preheated composites form a denser and more uniform polymer network with improved cross-linking under clinically relevant curing conditions [20]. The polymerisation behaviour of preheated composites is also dependent on light-curing parameters, indicating that composite temperature and curing mode interact to determine the final polymer structure and performance [20, 21].

Light-curing units (LCUs) differ in spectral output. Monowave devices emit within a narrow range optimised for camphorquinone, whereas polywave devices cover a broader spectrum to activate multiple photoinitiators [3, 10]. While polywave curing can improve polymerisation in certain materials, it may also create a less uniform polymer network, potentially reducing long-term optical stability [22].

Injectable composite resins—G-aenial Universal Injectable (nanohybrid) and Filtek Supreme Flowable (nanofilled)—are widely used with the injection moulding technique because of their thixotrophic property which facilitates easy penetration into the silicone mould and intimate adaptation to the mould space, allowing precise reproduction of tooth anatomy with minimal finishing and excellent polishability [23].

Despite these clinical advantages and their increasing use in esthetic restorative procedures, the colour stability of injectable composite resins—particularly under conditions simulating thermal and staining challenges—has not been extensively investigated in the literature [5, 18]. This study addresses this gap by evaluating the independent and interactive effects of preheating and curing mode on the colour stability of a nanohybrid (G-aenial Universal Injectable) and a nanofilled (Filtek Supreme Flowable) composite following coffee immersion.

The hypotheses tested were:

- H1: Preheating at 50 °C significantly affects colour stability compared with no preheating.
- H2: Polywave curing produces higher ΔE values than monowave curing.
- H3: There is a material-dependent interaction between preheating and curing mode.
- H4: Coffee immersion results in a clinically significant increase in ΔE across all groups.

## Materials and methods

### Study design and materials

This in vitro study assessed the effect of composite preheating and curing mode on the colour stability of two resin-based composites after coffee immersion. Eighty-disc shaped specimens were fabricated from:

- G-aenial Universal Injectable (GC Corporation, Tokyo, Japan)—nanohybrid, Bis-MEPP (Bis-EMA), UDMA, TEGDMA, silicon dioxide, strontium glass, 10–200 nm fillers, 69% wt / 50% vol.
- Filtek Supreme Flowable (3 M ESPE, St. Paul, MN, USA)—nanofilled, procrylat, Bis-GMA, TEGDMA, 20–75 nm fillers, 65% wt / 46% vol.

Two LED curing units were used: Monowave (420–480 nm, 1000–1700 mW/cm²) and Polywave (385–515 nm, 2300 mW/cm²). Output was verified with a radiometer [5, 10].

### Grouping and sample size

Sample size (*n* = 10/group) was calculated for α = 0.05, power = 0.80, and a 5-unit ΔE difference [24]. Each composite was assigned to four subgroups: non-preheated or preheated (50 °C), cured with monowave or polywave.

### Specimen fabrication

Specimens (10 mm × 2 mm) were prepared in a Teflon mould, with Mylar strips and glass slides to produce smooth surfaces [25, 26]. All sample fabrication was carried out by a single trained operator to ensure consistency. Preheated composites were warmed to 50 °C for 5 min before moulding [17, 18]. Polymerisation was performed per group: monowave for 20 s per side; polywave for 3 s per side.

The significantly shorter exposure time for the polywave unit was justified by its high irradiance output (2300 mW/cm²), which provides adequate energy delivery to achieve full polymerisation while minimizing thermal rise within the composite and surrounding environment [22]. Pilot verification using the radiometer confirmed stable irradiance output, and the energy dose delivered by the polywave unit exceeded the minimum requirement for effective polymer conversion. This ensured complete curing of the 2 mm-thick specimens without risking overheating or polymerization-related thermal stress [5, 10].

All specimens were light-cured under controlled environmental conditions (23 ± 1 °C and 50 ± 5% relative humidity) to eliminate temperature- or humidity-related variability during polymerisation. Standardised positioning was ensured by aligning each specimen perpendicular to the curing tip with a fixed distance of 1 mm, maintained using a custom positioning jig. The irradiance of both monowave and polywave LCUs was verified before each curing session using a calibrated radiometer to ensure output stability throughout the experiment, rather than only at baseline [19].

### Baseline colour measurement

After 24 h in distilled water at 37 °C, baseline L*, a*, b* values were recorded with a spectrophotometer (Spectraflash 600, Data Colour International, USA) under CIEL*a*b*. Three readings per specimen were averaged.

### Staining protocol

Coffee was prepared daily by dissolving 2.4 g powder in 200 ml boiling water, stirring 10 min, and filtering [19]. Specimens were immersed 3 h/day for 12 days at 37 °C, stored in distilled water between immersions.

### Post-staining colour measurement and analysis

Post-staining L*, a*, b* values were recorded, and ΔE calculated:

ΔE > 2.7 was deemed clinically perceptible [26]. Data were analysed using one-way ANOVA and Tamhane’s post hoc test (SPSS v.25, α = 0.05).

$$\Delta E = \sqrt {\left( {L_{2} - L_{1} } \right)^{2} + \left( {a_{2} - a_{1} } \right)^{2} + \left( {b_{2} - b_{1} } \right)^{2} }$$

## Results

### Overall findings

To strengthen statistical transparency, the assumptions of normality and homogeneity of variances were assessed prior to performing ANOVA.

Composite type, preheating, and curing mode each had a statistically significant effect on colour change (ΔE) after coffee immersion (*p* < 0.001). All groups recorded ΔE values above the clinical perceptibility threshold of 2.7, confirming that visible staining occurred under all tested conditions.

### Effect of composite type

Across all curing and preheating conditions, **G-aenial Universal Injectable** showed higher mean ΔE values than **Filtek Supreme Flowable** (*p* < 0.001), indicating greater staining susceptibility. Mean baseline and post-staining L*, a*, b* values, along with calculated ΔE, are presented in Table 1.

### Effect of curing mode

**Fig. 1:**
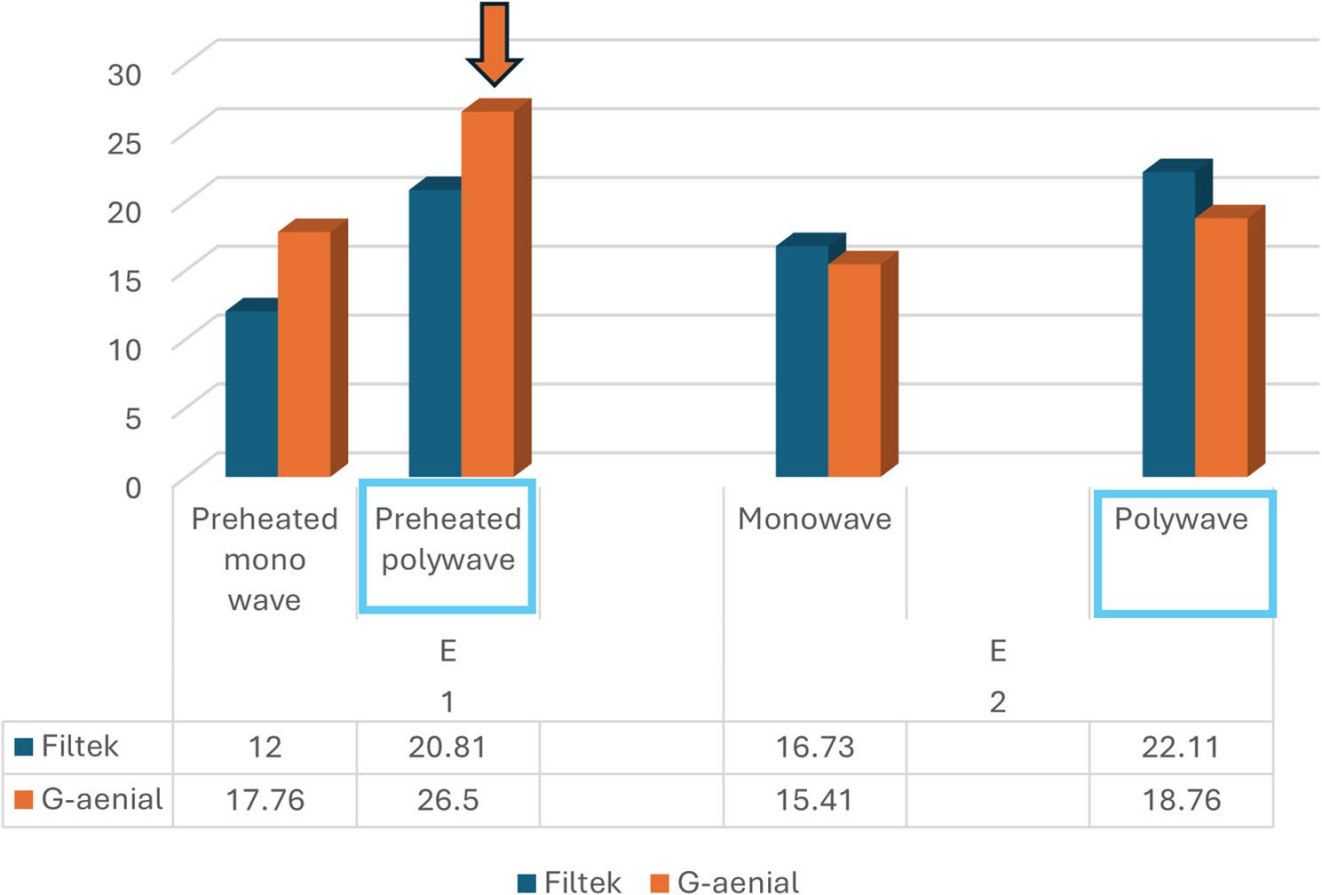
Illustrates mean ΔE across all eight subgroups, showing higher values for polywave curing. The orange arrow indicates the highest colour change value (ΔE) for GHP, also note the increase in (ΔE) for the polywave groups when compared to monowave groups

Polywave curing resulted in consistently higher ΔE values than monowave curing (*p* < 0.001). This difference was observed in both composites and under both preheating conditions. The bar graph in Fig. 1 visually compares ΔE values across the eight experimental groups, showing the general trend of greater discolouration in polywave-cured specimens (indicated using box).

### Effect of preheating

**Fig. 2:**
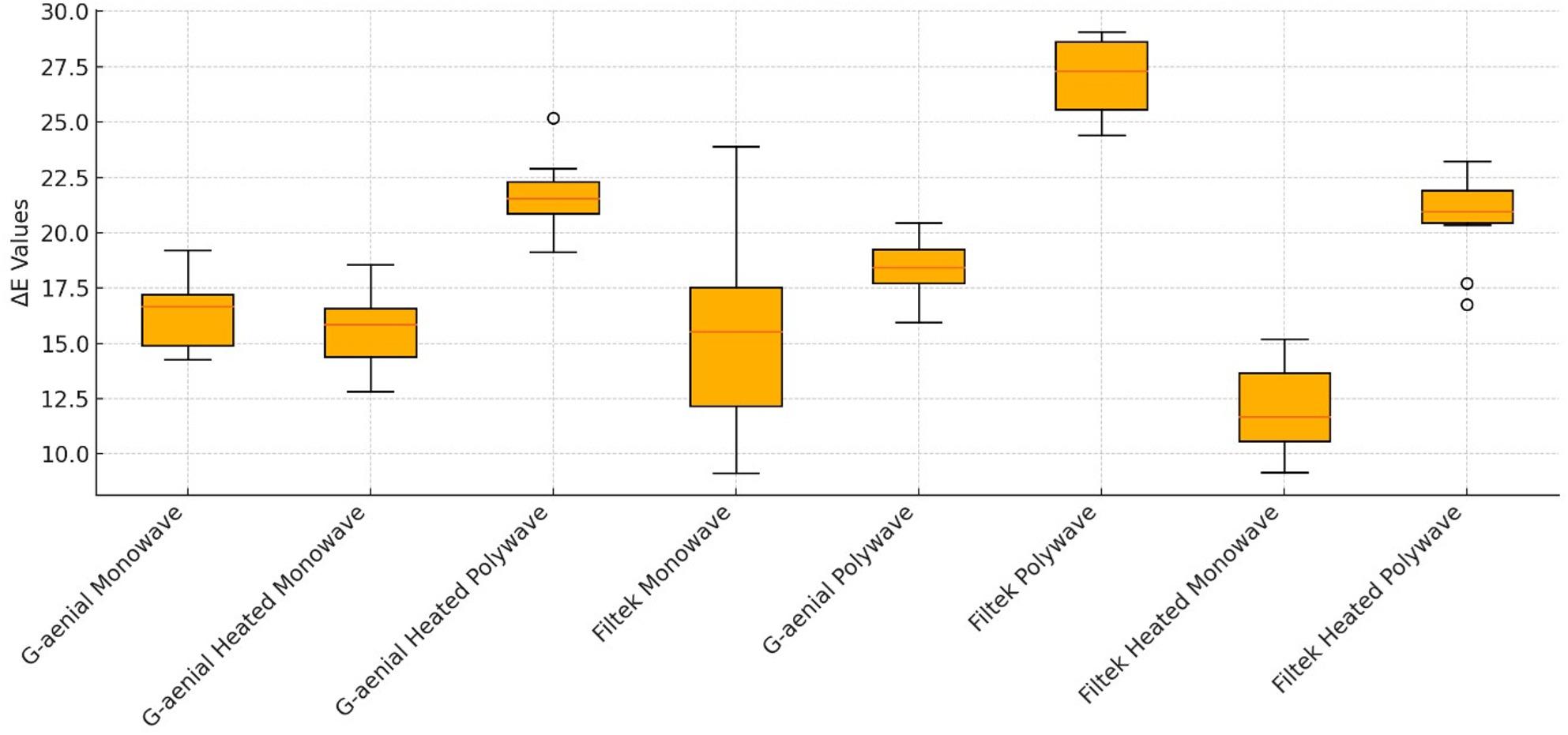
Depicts the distribution and variability of the ΔE values using box-and-whisker plots. The boxes represent the interquartile range with the median marked, while the whiskers indicate the spread of the data

Preheating at 50 °C increased ΔE values for both composites, with the largest effect in the G-aenial Polywave group, which recorded the highest mean colour change value of (ΔE = 26.50). Conversely, the Filtek Heated Monowave group had the lowest mean colour change value (ΔE = 12.0) of all tested conditions. The box plot in Fig. 2 illustrates ΔE distribution within each group, highlighting the broader range in preheated polywave groups compared to non-preheated monowave groups.

### Statistical comparisons

One-way ANOVA confirmed significant between-group differences in ΔE (F = 7.987, *p* < 0.001). Post hoc Tamhane’s multiple comparisons (Table 2) revealed:

- **Filtek polywave** had significantly higher ΔE than **G-aenial Monowave** (*p* = 0.001).
- **Filtek heated monowave** had significantly lower ΔE than **G-aenial Heated Polywave** (*p* = 0.012).
- Several additional significant pairwise differences were noted, further supporting that both composite composition and curing protocol influence colour stability.

### Clinical vs. statistical significance

Although statistically significant differences were identified between groups, all ΔE values exceeded 2.7, indicating clinically perceptible discolouration under all conditions. The fourth hypothesis (H4) was hence accepted as coffee staining had a significant impact on colour changes in all samples regardless of the tested groups. The data suggest that while appropriate selection of composite type and curing protocol can reduce staining, complete prevention is unlikely in high-pigment environments such as frequent coffee exposure (Fig. 1).

**Table 1:** Baseline and post-staining L*, a*, b* values and ΔE (mean ± SD) for each experimental group

Composite Type	Preheating	Curing Mode	L* (Baseline)	L* (Post)	a* (Baseline)	a* (Post)	b* (Baseline)	b* (Post)	ΔE ± SD
G-aenial Universal Injectable	No	Monowave	69.85 ± 1.00	59.39 ± 2.42	0.80 ± 0.27	6.36 ± 0.88	12.61 ± 1.05	22.47 ± 4.55	15.41
	No	Polywave	73.55 ± 0.70	61.50 ± 1.59	0.63 ± 0.40	6.99 ± 1.16	12.86 ± 1.32	25.75 ± 4.64	18.76
	Yes	Monowave	70.20 ± 1.25	56.07 ± 2.57	1.56 ± 0.52	7.70 ± 1.47	15.25 ± 1.37	24.09 ± 2.90	17.76
	Yes	Polywave	72.16 ± 1.55	60.90 ± 2.10	0.55 ± 0.29	6.96 ± 1.07	13.76 ± 2.84	31.68 ± 2.09	26.50
Filtek Supreme Flowable	No	Monowave	73.99 ± 1.22	62.30 ± 3.87	− 0.42 ± 0.18	4.85 ± 1.78	11.30 ± 2.07	22.05 ± 4.88	16.73
	No	Polywave	77.30 ± 0.76	61.80 ± 2.51	− 0.71 ± 0.06	7.19 ± 1.92	10.74 ± 0.94	30.73 ± 2.68	22.11
	Yes	Monowave	74.83 ± 1.03	66.27 ± 2.37	− 0.74 ± 0.27	4.04 ± 0.95	12.50 ± 1.54	19.42 ± 5.99	12.00
	Yes	Polywave	77.05 ± 1.01	65.44 ± 1.55	− 0.74 ± 0.13	3.64 ± 0.87	11.60 ± 0.58	28.30 ± 2.43	20.81

ΔE calculated using CIELab formula; higher values indicate greater colour change*

*SD* standard deviation

**Table 2:** Statistically significant pairwise comparisons of ΔE (Tamhane’s post hoc test)

Comparison (Group i vs. Group j)	Mean difference (ΔE)	95% CI (Lower, Upper)	*p*-value
GM vs. FP	− 10.88	− 18.22, − 3.53	0.001
GHM vs. FP	− 8.61	− 16.38, − 0.84	0.021
GHP vs. FHM	9.68	1.51, 17.85	0.012
FM vs. FP	− 9.70	− 18.83, − 0.58	0.031
GP vs. FP	− 7.62	− 15.12, − 0.12	0.044
FP vs. FHM	14.10	5.90, 22.29	< 0.001
FHP vs. FHM	8.39	0.86, 15.93	0.022

Only statistically significant differences (p < 0.05) are shown

Positive mean difference indicates Group i had higher ΔE; negative indicates Group j had higher ΔE

*GM *G-aenial Monowave,* FP*  Filtek polywave,* GHM* G-aenial Heated Monowave,* GHP* G-aenial Heated Polywave,* FHM* Filtek Heated Monowave,* FM*  Filtek Monowave, * GP * G-aenial Polywave, * FHP*  Filtek Heated Polywave

## Discussion

This study examined how composite type, preheating, and curing mode—independently and in combination—affect colour stability after immersion in coffee solution. The findings clearly demonstrate that all three variables significantly influence the degree of discolouration, with results showing both statistically and clinically relevant differences among experimental groups.

### Influence of composite type

Across all conditions, G-aenial Universal Injectable exhibited greater ΔE values than Filtek Supreme Flowable (Table 1; Fig. 1), confirming its higher susceptibility to staining. These results align with Uctasli et al. [1], who reported that nanohybrid composites tend to discolour more than nanofilled counterparts due to variations in resin matrix content and filler distribution. Paolone et al. [2] also emphasised that higher resin content increases the potential for pigment absorption. The nanohybrid’s larger filler size range and slightly lower filler–resin interface density may facilitate deeper chromogen penetration compared to the more densely packed nanoparticle arrangement in nanofilled composites [5, 6, 17].

### Effect of curing mode

The second hypothesis (H2) was accepted as polywave curing consistently produced higher ΔE values than monowave curing (Fig. 1), regardless of composite type or preheating condition. This observation is in agreement with Dumitrescu et al. [14], who reported that broader spectral output can create heterogeneity in the polymer network, potentially increasing water sorption and pigment diffusion. While polywave units are designed to activate multiple photoinitiators [3, 10], the resulting polymerisation profile may be less uniform, particularly in materials optimised for narrower wavelength curing, thereby reducing long-term optical stability.

### Impact of preheating

Preheating significantly increased ΔE in both composites, with the effect most pronounced in the G-aenial Polywave group, which recorded the highest mean ΔE of the study (Fig. 2; ΔE = 26.5). This supports findings by Mundim et al. [18] and Sahu and Gupta [27], who suggested that while preheating enhances polymerisation through greater monomer mobility, it can also alter the polymer network, creating pathways that facilitate water uptake and pigment ingress. Starkova et al. [28] further showed that water diffusion through nanoparticle-filled matrices can promote discolouration via interfacial degradation between filler and resin matrix. The first hypothesis (H1) was accepted, as preheating to 50 degrees led to significant difference in color stability.

Interestingly, the Filtek Heated Monowave group recorded the lowest ΔE of all groups (Table 1; ΔE = 12), indicating that in certain composite–curing mode combinations, preheating may not only be benign but potentially beneficial for colour stability. This finding warrants further investigation to determine whether the observed effect is material-specific or more broadly applicable.

### Interaction between preheating and curing mode

The significant interaction effects identified in Table 2 suggest that the influence of preheating on colour stability is not uniform across curing modes or materials. The combination of preheating and polywave curing appeared particularly detrimental for G-aenial, likely due to compounded effects of thermally induced network changes and the broader spectral curing profile. Conversely, when preheating was paired with monowave curing in Filtek, colour stability was enhanced, perhaps due to improved network homogeneity and optimal activation of the material’s primary photoinitiator. The third hypothesis (H3) was accepted as both the composite types interacted distinctly and independently to preheating and to the applied curing modes.

### Clinical relevance

This study shows that both preheating and light-curing mode have a measurable impact on the long-term optical stability of resin composites. Although warming the composite before placement can improve handling and promote greater monomer conversion, it may also heighten staining susceptibility—particularly when combined with polywave curing. Discolouration varied by material; the nanohybrid composite (G-aenial) showed greater colourchange than the nanofilled composite (Filtek) under comparable conditions. These findings underscore the importance of considering material-specific interactions between resin formulation, curing spectrum, and preheating. Clinicians should weigh the immediate handling benefits of preheating against its potential aesthetic drawbacks, selecting protocols that achieve both functional longevity and sustained visual appeal. Additionally, maintenance measures such as routine polishing or surface glazing could mitigate pigment accumulation [29].

## Limitations and future directions

This study was conducted under controlled in vitro conditions that do not fully replicate the oral environment. Factors such as salivary flow, enzymatic activity, and dietary variability may alter the staining process [11]. Only coffee was tested; other beverages such as tea, red wine, and cola may produce different staining profiles [2]. The study was also limited to two composites and two curing units, which restricts generalisability to all commercially available materials. Additionally, the 12-day immersion period, although commonly accepted as equivalent to approximately one clinical year of staining exposure, represents an accelerated aging model and may not fully replicate cumulative long-term intraoral challenges [30]. Furthermore, surface roughness was not evaluated following immersion. Since alterations in surface texture can influence both stain adherence and the visual perception of color change, the absence of this assessment limits interpretation of the staining mechanism [31].

Future research should explore multiple staining agents, include additional composite chemistries and photoinitiator systems, and conduct long-term in vivo studies to validate laboratory findings. Advanced analytical techniques such as FTIR spectroscopy and scanning electron microscopy could further elucidate the structural changes induced by preheating and varying curing spectra [14].

## Conclusion

Composite formulation, preheating, and curing mode each significantly influenced colour stability after coffee immersion. The nanohybrid composite (G-aenial Universal Injectable) showed greater staining than the nanofilled composite (Filtek Supreme Flowable). Polywave curing generally produced higher ΔE values than monowave curing, and preheating increased discolouration in most cases—most notably in the G-aenial Polywave group—while the Filtek Heated Monowave group showed the lowest ΔE.

Clinically, choosing a nanofilled composite, avoiding unnecessary preheating in aesthetic zones, and using monowave curing may help reduce staining risk, though all tested conditions exceeded the perceptibility threshold. Preventive maintenance and patient education remain essential. Further in vivo research is recommended to confirm these findings and refine clinical protocols.

## Data Availability

“The datasets used and/or analysed during the current study are available from the corresponding author on reasonable request.”

## Abbreviations

ANOVA: Analysis of variance
GM: G-aenial Monowave
FP: Filtek polywave
GMH: G-aenial Heated Monowave
GHP: G-aenial Heated Polywave
FHM: Filtek Heated Monowave
FM: Filtek Monowave
GP: G-aenial Polywave
FHP: Filtek Heated Polywave
LCU: Light Cure Unit
